# Correction Notice: Coronavirus Disease 2019 in Recipient of Allogeneic Hematopoietic Stem Cell Transplantation: Life-threatening Features Within the Early Post-engraftment Phase

**DOI:** 10.1097/HS9.0000000000000493

**Published:** 2020-09-16

In the article entitled “Coronavirus Disease 2019 in Recipient of Allogeneic Hematopoietic Stem Cell Transplantation: Life-threatening Features Within the Early Post-engraftment Phase” (*HemaSphere*. 2020;4:e448), there was an error in the original published title. “Life-threatening” instead appeared as “Life-threating.” The title has now been corrected online:

https://journals.lww.com/hemasphere/Fulltext/2020/08000/Coronavirus_Disease_2019_in_Recipient_of.14.aspx

